# Expression profiles of immune mediators in feline Coronavirus-infected cells and clinical samples of feline Coronavirus-positive cats

**DOI:** 10.1186/s12917-017-1019-2

**Published:** 2017-04-07

**Authors:** Nikoo Safi, Amin Haghani, Shing Wei Ng, Gayathri Thevi Selvarajah, Farina Mustaffa-Kamal, Abdul Rahman Omar

**Affiliations:** 1 Institute of Bioscience, Universiti Putra Malaysia, Serdang, Selangor Malaysia; 2 Faculty of Veterinary Medicine, Universiti Putra Malaysia, Serdang, Selangor Malaysia; 3 Leonard Davis School of Gerontology, University of Southern California, Los Angeles, CA USA

**Keywords:** Feline Coronavirus, Cytokine, Immune-mediators

## Abstract

**Background:**

There are two biotypes of feline coronavirus (FCoV): the self-limiting feline enteric coronavirus (FECV) and the feline infectious peritonitis virus (FIPV), which causes feline infectious peritonitis (FIP), a fatal disease associated with cats living in multi-cat environments. This study provides an insight on the various immune mediators detected in FCoV-positive cats which may be responsible for the development of FIP.

**Results:**

In this study, using real-time PCR and multiplex bead-based immunoassay, the expression profiles of several immune mediators were examined in Crandell-Reese feline kidney (CRFK) cells infected with the feline coronavirus (FCoV) strain FIPV 79–1146 and in samples obtained from FCoV-positive cats. CRFK cells infected with FIPV 79–1146 showed an increase in the expression of interferon-related genes and pro-inflammatory cytokines such as MX1, viperin, CXCL10, CCL8, RANTES, KC, MCP1, and IL8. In addition, an increase in the expression of the above cytokines as well as GM-CSF and IFNγ was also detected in the PBMC, serum, and peritoneal effusions of FCoV-positive cats. Although the expression of MX1 and viperin genes was variable between cats, the expression of these two genes was relatively higher in cats having peritoneal effusion compared to cats without clinically obvious effusion. Higher viral load was also detected in the supernatant of peritoneal effusions compared to in the plasma of FCoV-positive cats. As expected, the secretion of IL1β, IL6 and TNFα was readily detected in the supernatant of peritoneal effusions of the FCoV-positive cats.

**Conclusions:**

This study has identified various pro-inflammatory cytokines and interferon-related genes such as MX1, viperin, CXCL10, CCL8, RANTES, KC, MCP1, IL8, GM-CSF and IFNγ in FCoV-positive cats. With the exception of MX1 and viperin, no distinct pattern of immune mediators was observed that distinguished between FCoV-positive cats with and without peritoneal effusion. Further studies based on definitive diagnosis of FIP need to be performed to confirm the clinical importance of this study.

## Background

Feline coronavirus (FCoV) can be divided into two biotypes: the ubiquitous feline enteric coronavirus (FECV) which often causes self-limiting diarrhea, and the feline infectious peritonitis virus (FIPV), the mutated form which causes fatal disease in cats [1, 2]. The widely accepted ‘internal mutation’ theory describes that mutations in FECV give rise to FIP de novo. In addition, it was suggested that these mutations occur in the monocytes, rather than the intestinal epithelial cells where the FECV first enters the host [3, 4]. FCoV travels to organs and tissues through monocyte-associated viremia where it is later disseminated in the endothelial venules of the serosa, omentum, pleura, meninges and uveal tract (reviewed in [1, 2]).

Currently, there are no specific markers to distinguish the two biotypes, thus making the diagnosis of feline infectious peritonitis (FIP) difficult. Although several studies have reported several point mutations in the S gene that are associated with occurrence of FIPV, it remains unclear whether the mutations contributed solely to the development of FIP [5–7]. Therefore, ante-mortem confirmation of FIP remains a challenging task in clinical research of FIP.

Information on the immunopathogenesis and the role of cytokines, and immune mediators in FCoV infection are relatively sparse. Although it is generally known that FECV causes self-limiting disease, cats can become persistent shedders contributing to the transmission of the disease (reviewed in [1, 2]). However, only approximately 5% of cats harboring FECV actually develop FIP [1, 8]. The exact nature of this immunity is still unknown although the development of FIP has been postulated to correlate with the magnitude of immune responses, as cats with robust cell-mediated immune (CMI) response have been found to resist the disease [9]. In contrast, humoral response does not seem to be beneficial and could lead to the dissemination of the virus through complement activation via formation of immune complexes and vasculitis associated with type III hypersensitivity (reviewed in [1, 2]). This would then lead to effusive FIP (wet form), the most commonly reported form of FIP due to the obvious sign of peritoneal effusion. The non-effusive form of FIP is associated with partial CMI response in the individual cat to contain the virus leading to the formation of granulomas containing macrophages, which could then be replaced by B cells and plasma cells [10, 11].

To date, there are no specific immune markers that could distinguish FECV from FIPV infections. However, the observed cytokine patterns are different between asymptomatic FCoV-infected cats and those with clinical signs of FIP [12]. Asymptomatic FCoV-infected cats generally show higher IL10 in the spleen, suggesting the ability to control excessive inflammation triggered by macrophages. Furthermore, lymphocyte depletion has been indicated as one of the hallmarks of FIP and postulated to be induced by excessive production of TNFα [13–15]. In contrast, high IFN γ and IL 1β production has been associated with protection against FIP [16]. Increase in Th1-like cytokines such as IL12/p40 and IFNγ, which were associated with the decrease of IL4 in the lymphoid tissue, has been observed in cats experimentally infected with FIPV [13]. Furthermore, previous studies showed deregulation of different mediators, illustrated by the upregulation of pro-inflammatory cytokines such as IL1β, IL6, TNFα, MIP1α, RANTES, and IFNγ in peritoneal effusions and serum samples of FIP clinical cases [17–19].

Recently, we used a transcriptomic approach by next-generation sequencing of RNA from Crandell-Reese feline kidney (CRFK) cells infected with the FCoV strain FIPV 79–1146 to elucidate the complex interaction between the virus and host cells in vitro [20, 21]. Results revealed that, during the first 3 h of infection, at least 96 transcripts associated with immune responses (e.g. ISGs, MX1, RSAD2, A3C, ID1, CRIP1, TRIM25 and MDA5), apoptosis (ID1, ATF3, TNFα, and RNF7), and pro-inflammatory responses (e.g. PD-L1, CCL8, CXCL10 and CCL17) were downregulated. Only a few genes, namely PD-1, PD-L1 and A3H, has been previously characterized in a study on FCoV-infected CRFK cells and expression profiles in peripheral blood mononuclear cells (PBMC) of cats diagnosed with FIP [20]. Characterization of additional immune mediators that modulate innate and acquired immune responses will increase our understanding of their involvement during FIPV infection. The objective of this study was to investigate the immune mediator profiles in CRFK-infected cells and FCoV-positive cats. Both gene and protein expression profiles were determined by quantitative real-time PCR (qPCR) and multiplex bead-based assays.

## Methods

### In vitro analysis of FCoV-infected cells

TCID50 of the FCoV strain FIPV 79–1146 (ATCC® VR2202) [22] was determined using endpoint dilution assay. Virus infectivity was confirmed by RT-PCR (Bioline, UK) detecting the FCoV conserved 3′ untranslated region (3′-UTR) [23]. To prepare a sufficient amount of infected cells at different time points, two confluent 75 cm^2^ flasks of CRFK cells (ATCC® CCL-94™) were inoculated at each time point with 3 ml TCID_50_/ml (MOI = 0.1) of FIPV 79–1146 and the virus inoculum was left in the culture. At 3, 12, 24, 48 and 72 h post-inoculation (hpi), the cells were trypsinized and cell pellets were collected upon centrifugation. The uninfected flask was designated as 0 hpi. The cell pellets were stored at −80 °C until further use for virus and immune mediator detection by real-time PCR and multiplex bead-based immunoassay.

### Selection criteria for FCoV-positive cats

Before performing the in vivo phase of the study, approval for handling and sampling cats was obtained from the Institutional Animal Care and Use Committee (IACUC), Faculty of Veterinary Medicine, Universiti Putra Malaysia (UPM) with the reference number UPM/IACUC/AUP-R040/2014. The status of FCoV infection was evaluated in cats that were presented to the University Veterinary Hospital (UVH), UPM, using Biogal’s Immunocomb Antibody Test Kit (Biogal-Galed Laboratories, Israel) to determine the antibody titer, followed by reverse transcriptase quantitative PCR (RT-qPCR) to detect the presence of FCoV in the serum [23]. Combscale S value was used as a colorimetric indicator for the determination of anti-FCoV antibody titer, where cats with antibody titers ≥ S2+ were chosen for further analysis [24]. In addition, cats were also screened serologically for Feline Immunodeficiency Virus (FIV) and Feline Leukemia Virus (FELV) using the SNAP FIV/FELV Combo test (IDEXX Laboratories, USA) according to the manufacturer’s protocol. Cats with high antibody levels against FCoV (titer ≥ S2+) and that are seronegative for FIV and FELV were selected and further underwent hematology evaluation. In addition to that, the presence or absence of peritoneal effusion was also evaluated in these selected cats. Post-mortem examination and follow-up analysis were not carried out to arrive at definitive diagnosis of FIP. Healthy seronegative FIV/FELV cats with absence of antibody titer against FCoV were considered as negative control cats.

### Blood collection for preparation of PBMC and plasma

A total of 2.5 ml blood was collected from FCoV antibody titer ≥ S2+, FIV- and FELV- cats. The collected blood samples were immediately divided into two tubes for different purposes. First, 0.5 ml of blood was stored in clot activator tubes (BD Vacutainer® Tubes with BD hemoguard closure, USA) on ice and kept at 4 °C for serum separation. The remainder of the blood was transferred into EDTA tubes (BD Vacutainer® Tubes with BD hemoguard closure, USA) for PBMC isolation and plasma collection. The collected serum was stored at −80 °C for multiplex bead-based immunoassay. Isolation of PBMC was performed using Ficoll-Paque PLUS (GE Healthcare Life Science, USA) following the steps provided by the manufacturer. Plasma and PBMC were collected separately and stored at −80 °C until further use in real-time PCR for measuring viral load and mRNA expression of immune-related genes.

### Peritoneal effusion

Peritoneal effusion (PE) samples were collected from FCoV-positive cats and centrifuged at 400×g for 10 min at 4 °C. The obtained cell pellets were used for detecting expression of immune-related genes using real-time PCR, whereas the supernatants were used for virus detection using RT-qPCR and for measuring cytokine and chemokine levels using multiplex bead-based immunoassay.

### RNA extraction

Cellular RNA was extracted from the CRFK, PBMC and PE cells using the RNeasy Mini Kit, which includes DNase treatment (Qiagen, Germany), following the protocol supplied by the manufacturer. Viral RNA was extracted from the cell culture pellet, plasma, and supernatant of the PE (PES) using the Viral Nucleic Acid Extraction Kit 2 (Geneaid, Taiwan) according to the manufacturer’s instructions. The concentration and quality of the extracted RNA were analyzed using a BioSpectrometer (Eppendorf, Germany). The extracted RNA samples (100 ng/μl) were used immediately to synthesize cDNA or kept at −80 °C for future usage.

### Detection of viral load by SYBR green-based real-time PCR

cDNA was synthesized using the SensiFAST™ cDNA Kit (Bioline, UK), as instructed by the manufacturer. Virus quantification was performed using SYBR Green-based real-time PCR as described previously with a slight modification [25]. Briefly, quantitative real-time PCR (qPCR) was performed in a 20 μl reaction consisting of 1 μl cDNA, 1 μl forward primer (1 μmol), 1 μl reverse primer (1 μmol), 7 μl nuclease-free water and 10 μl 2× SensiFAST SYBR® No-ROX mix (Bioline, UK). The qPCR reaction was performed using the CFX96 Touch TM Real-Time PCR Detection System (Bio-Rad, USA) with the following cycling conditions: one cycle at 95 °C for PCR activation and 40 cycles of denaturation at 95 °C for 5 s, annealing at 60 °C for 10 s, and extension at 70 °C for 20 s. Detection of viral load was done by absolute quantification based on a standard curve generated from the serial dilution of a cDNA template. Viral load was expressed as viral copy number following a formula described previously [26].

### Detection of immune-related mRNA expression by TaqMan-based real-time PCR

The expression of five immune-related genes, namely CCL8 (MCP2), viperin (RSAD2), CXCL10, MX1, and CCL17, and one reference gene (GAPDH) was measured by TaqMan-based real-time PCR (qPCR). The forward primers, reverse primers and TaqMan MGB probes were designed based on the *Felis catus* genome sequence [27]. The sequences of the primers and probes were designed using the CLC genomic workbench software, while primer characteristics were analyzed using Primer3 (http://bioinfo.ut.ee/primer3-0.4.0/) and Basic Local Alignment Search Tool (BLAST) to confirm alignment with more than 80% of the related gene in the *Felis catus* genome (Applied Biosystem, USA) (Table 1). cDNA was prepared using the Tetro cDNA Synthesis Kit (Bioline, UK) according to the manufacturer’s protocol with a slight modification, in which specific forward and reverse primers for each gene were used instead of random hexamers. The RNA extracted from FIPV-infected CRFK cells at 48 hpi was used to optimize the real-time PCR assay before the assay was used to measure expression in the clinical samples obtained from FCoV-positive cats. Using serially diluted cDNA of each gene, the designed primer sets produced specific amplification with high PCR efficiency. Furthermore, primers for each gene were designed spanning two different exons to ensure specificity.

**Table 1:** TaqMan primers, MGB probes and accession numbers of the analyzed immune-related genes

Gene	Primers sequences	MGB Probe	Accession number	Annealing temperature (°C)
CCL8	117CTTGCTCAGCCAGGTTCAGTT137 183GGATCTTCCCTTTGACCACACT162	6FAMCCATCCCAATTACCTGCTMGBNFQ	XM_003996558	66
Viperin	219CCCCCACCAGCGTCAAC235 281GGAAGCAGAAGCCACACTTGT261	6FAMACCACTTCACCCGCCAGMGBNFQ	XM_003984516	60
CXCL10	332ACACAGAAGCATAATCACCGTACTG356 399GGGAAATGATGGCAGAGGTAGT378	6FAMCAAAGATGGACCAGAAAGMGBNFQ	XM_003985274	60
MX1	469CAGGACTTTGAGACGGAGATTTC491 535CATTCTGGGCTGTATTGATTGC514	6FAMCCCTTCGGAGGTGGAMGBNFQ	XM_006935851	60
CCL17	119GGGCCATCCCTCTCAGAAG137 189CACTATGGCGTCTTTGGAACACT167	6FAMTGACAGGGTGGTACAGGAMGBNFQ	NM_001009849	60
GAPDH^a^	71GTCCCCGAGACACGATGGT89 130CCAGGCGCCCAATACG115	6FAMAAGGTCGGAGTCAACGGMGBNFQ	XM_006933438	57

Note: ^a^Reference gene

qPCR was performed using the TaqMan Fast Advanced Master Mix (Life Technologies®, Applied Biosystems, USA) according to the manufacturer’s protocol. 20 μl reactions were prepared as follows: 1 μl cDNA template, 0.5 μl forward primer (450 ηM), 0.5 μl reverse primer (450 ηM), 1 μl probe (250 ηM), 7 μl nuclease-free water and 10 μl of Fast Advanced Master Mix. The Taqman Fast Advanced Master Mix consisted of AmpliTaq Fast DNA Polymerase, Uracil-N-glycosylase (UNG), dNTPs with dUTP, ROX dye, and optimized buffer components. RT-qPCR was performed on the CFX96 Touch TM Real-Time PCR Detection System (Bio-Rad, USA) with the following steps: initial UNG incubation at 50 °C for 2 min and PCR activation at 95 °C for 20 s, followed by 40 cycles of denaturation at 95 °C for 5 s, annealing at optimized temperature for 10 s (Table 1), and extension at 72 °C for 20 s. The PCR efficiency of GAPDH, CXCL10, MX-1, viperin, CCL17 and CCL8 was 100, 99, 101, 102, 100 and 100%, respectively. For data interpretation, relative expression analysis (ΔΔCq) followed by analysis of variance (ANOVA) (*p* < 0.05) were carried out to determine the expression changes of target genes across different time points. Relative expression of the different immune-related genes were normalized to GAPDH and the negative controls.

### Detection of immune-related protein expression by multiplex bead-based immunoassay

Measurement of 19 different immune-related protein expression was performed using the feline cytokines/chemokine magnetic bead-based panel immunoassay, FCYTOMAG-20 K FCYTMAG-20 K-PMX (MILLIPLEX MAP Kit, EMD Millipore Corporation, USA) following the manufacturer’s instructions. The assay’s principle of quantitative analysis was based on the standard provided in the kit. The standard was a mixture of all immune-related proteins at certain concentrations prepared by dilution as described by the kit. Hence, the concentrations of immune-related proteins in the samples were measured using the standard curve generated by the standard. The prepared incubated plates (containing samples, standard and quality controls) were read on a Luminex analyzer (MAGPIX). Data obtained from the analyzer were analyzed by the MILLIPLEX analyst v5.1 software using five parameters logistic regression (EMD Millipore).

### Statistical analysis

Data generated from this study were represented as means ± standard error of the mean (SEM). Statistical package for the social sciences (SPSS) version 22 was used to perform factorial analysis of variance (ANOVA) at 0.05 levels of significance for both the in vitro and in vivo experiments. Duncan test was used for post hoc analysis between the groups.

## Results

### Detection of viral load

Viral load in the infected cells was detected based on the 3′ UTR region of FIPV using SYBR green-based real-time PCR. An increase in viral load was detected at different time points, with the peak viral load of 10^12.54^ occurring at 48 hpi, while the lowest viral load was detected at 3 hpi (Table 2). Total RNA obtained from the CRFK cells at 72 hpi was used to optimize the real-time PCR. The real-time PCR assay has a PCR efficiency of 100%.

**Table 2:** Intracellular FCoV load in CRFK cells at different time points post infection

Time points (hpi)	FCoV copy number [Mean ± SEM (log10)]
	Intracellular
0^a^	-
3^b^	5.22 ± 0.12
12^d^	10.28 ± 0.06
24^c^	6.33 ± 0.02
48^f^	12.54 ± 0.34
72^e^	11.83 ± 0.05

Note: Different alphabets indicate significant difference (*p* < 0.05) following Duncan post hoc analysis of three replicates from three independent experiments

### Expression profiles of immune-related genes in FIPV 79–1146-infected cells

All the analyzed immune-related genes showed significant (*p* < 0.05) changes in expression levels at different time points following infection with FCoV strain FIPV 79–1146. These genes were selected based on transcriptome data from our previous study on CRFK cells infected with FIPV 79–1146 [14]. In this study, we confirmed the upregulation of these genes at 3 hpi using Taqman real-time PCR. CCL8 and MX1 showed peak expression levels at 48 hpi, while CXCL10 and viperin showed the highest expression at 72 hpi (Table 3). Although the expression of viperin was upregulated at 48 and 72 hpi, its expression was downregulated at 3 and 12 hpi (Table 3).

**Table 3:** Relative expression of immune-related genes following FIPV 79–1146 infection of CRFK cells

Time points (hpi)	CCL8 (MCP2)	CXCL10 (IP10)	CCL17	MX1	Viperin (RSAD2)
0	1 ± 0^a^	1 ± 0^a^	1 ± 0^a^	1 ± 0^a^	1 ± 0^c^
3	21.67 ± 0.57^d^	13,341.2 ± 197.75^c^	39.68 ± 1.61^e^	4.49 ± 0.62^c^	−13.04 ± 0^a^
12	3.41 ± 0.07^b^	8712.95 ± 343.29^b^	3.56 ± 0^b^	9.16 ± 1.27^d^	−7.82 ± 0.02^b^
24	4.92 ± 0.04^c^	1,835,241.44 ± 7662.16^d^	22.87 ± 0.69^d^	2.69 ± 0.11^b^	5.72 ± 0.03^d^
48	40,322.18 ± 14.38^f^	8,569,241.92 ± 44,483.37^e^	39.86 ± 0.6^e^	900.72 ± 4.25^f^	353.53 ± 1.82^e^
72	21,651.02 ± 510.17^e^	8,776,535.79 ± 30,986.02^e^	8.51 ± 0.44^c^	517.06 ± 5.38^e^	583.3 ± 9.86^f^

Note: Data are presented as means ± SEM of three replicates from two independent experiments. Different alphabets above the data indicate significant difference following Duncan post hoc comparison of each column (*p* < 0.05). Relative expression (ΔΔCq) was calculated by normalizing with the reference gene (GAPDH) and the negative controls

### Detection of immune-related proteins in FIPV 79–1146-infected CRFK cells

A total of 19 different immune-related proteins were analyzed by a bead-based multiplex immunoassay at different time points, following infection with FIPV 79–1146. The panel of proteins was chosen since it comprised of mediators with known functions in antiviral immunity, modulation of pro-inflammatory responses and regulation of viral-induced apoptosis. Out of the 19 immune-related proteins, only IL8 (CXCL8), KC (CXCL1), RANTES (CCL5) and MCP1 (CCL2) were detected in the CRFK-infected cells (Table 4). We were unable to detect the expression of other proteins, most likely due to the non-hematopoietic origin of CRFK cells whereby they did not secrete the proteins and/or the expression levels were too low beyond the detection limit of the assay.

**Table 4:** Measurement of immune-related protein concentrations (pg/ml) in FIPV 79–1146-infected CRFK cells at different time points

Time points (hpi)	CXCL8 (IL8)	CXCL1 (KC)	CCL5 (RANTES)	CCL2 (MCP1)
0	465.33 ± 2.14^b^	9.23 ± 0.02^a^	22.63 ± 0.27^c^	913.03 ± 0.005^a^
3	166.75 ± 25.74^a^	28.55 ± 9.21^b^	8.35 ± 0.004^a^	960.84 ± 0.005^b^
12	444.32 ± 3.03^b^	8.32 ± 0.01^a^	31.18 ± 0.61^d^	923.22 ± 0.004^a^
24	1564.5 ± 45^c^	151.55 ± 9.98^c^	16.75 ± 0.73^b^	994.92 ± 7.38^b^
48	1499.5 ± 82.34^c^	119.02 ± 12.54^c^	2470 ± 114.67^f^	1068.5 ± 41.67^c^
72	2551 ± 93.33^d^	334.83 ± 9.49^d^	126.6 ± 2.4^e^	1050 ± 0.001^c^

Note: Data are represented as means ± SEM of three replicates from two independent experiments. Different alphabets above the data indicate significant difference following Duncan post hoc comparison of each column (*p* < 0.05). Peak expression levels of the cytokines were detected at 48 and/or 72 hpi

FIPV infection of CRFK cells caused a significant modulation in the expression of the detectable cytokines, with peak expression detected at 48 hpi (CCL2 and CCL5) or 72 hpi (CXCL1 and CXCL8). However, CXCL8 and CCL5 were downregulated at 3 hpi (*p* > 0.05). CCL2 showed the least changes in expression compared to other cytokines following FIPV 79–1146 infection.

### Detection of immune-related protein expression in FCoV-positive cats

#### Clinical features of the cats

The sampling of FCoV-positive cats was carried out at the University Veterinary Hospital-Universiti Putra Malaysia (UVH-UPM) over 1 year. Out of 150 cats, a total of 15 cats of different sex, age and breed that tested positive for high (≥ S + 2) FCoV antibody titer and FCoV RNA by RT-PCR but negative for FELV and FIV antibodies were considered for this study (Table 5). In addition, among the 15 FCoV-positive cats, nine cats were presented with peritoneal effusions, hence they were categorized into the effusive cohort. The remaining six cats were either asymptomatic (cat 6, 14 and 15) or having signs associated with non-effusive FIP (cat 2, 3 and 16) (Table 5).

**Table 5:** Demographic and clinical features of the cats considered for this study

ID	Age	Sex	Breed	FCoV titer	FELV /FIV titer	Body temperature °C	Peritoneal effusion	A:G ratio
1	2–4 years	F	DSH	0	−	−	−	NA
2	1 year	M	Persian	S2+	−	N/A	−	0.6
3	7 months	M	Persian	S3+	−	N/A	−	NA
6	8 months	F	DSH	S5+	−	N/A	−	NA
14	2 years	M	DSH	S5+	−	N/A	−	NA
15	2 years	F	DSH	S5+	−	N/A	−	NA
16	8 months	F	Persian	S5+	−	37.9	−	0.3
4	2 years	M	DSH	S3+	−	N/A	+	NA
7	7 months	M	Maine coon	S5+	−	37.3	+	0.3
8	9 months	M	DSH	S4+	−	39.2	+	0.4
9	3 years	M	DSH	S5+	−	38.6	+	0.6
5	2 years	M	DSH	S5+	−	39.8	+	0.3
10	8 months	M	DSH	S5+	−	N/A	+	0.3
11	10 months	M	Maine coon	S4+	−	40.5	+	0.3
12	1 year	M	Maine coon	S4+	−	38.3	+	0.5
13	11 months	M	Persian	S5+	−	40.0	+	0.4

Note: *NA* not available, *DSH* Domestic short hair, *A:G* Albumin/Globulin, *F* Female, *M* Male, *FCoV scoring of S2+ titer* low positive reaction, *≥S3+ titer* positive reaction, *≥S5+ titer* high positive reaction

Hematology examination of the nine cats with effusions showed evidence of thrombocytopenia, hyperbilirubinemia, hyperglobulinemia and hypoalbuminemia, with three of these cats also having lymphopenia and icterus. In addition, the cats had albumin/globulin (A: G) ratios of between 0.3–0.6. Cat 1 represents three healthy FCoV-negative cats aged 2–4 years that also tested negative for FIV and FELV antibodies.

#### Detection of FCoV load in FCoV-seropositive cats

FCoV was quantified by RT-qPCR in blood plasma and supernatant of the peritoneal effusion (PES) taken from the FCoV-seropositive cats. All of the cats, except for the FCoV-seronegative cats, had positive viral load in the plasma and PES (Table 6). Furthermore, the level of viral load in the PES was significantly higher (*p* < 0.05) than in the plasma for the majority of the cats (Table 6). Only two cats (cats 10 and 11) exhibited higher viral load in the plasma. Almost all cats with peritoneal effusions had higher plasma viral load (*p* < 0.05) compared to cats without peritoneal effusion.

**Table 6:** Detection of FCoV load in plasma and supernatant of peritoneal effusion

Cat status	Cat ID	FCoV copy number [Mean ± SEM (log10)]	
		Plasma	PES
Negative controls	1^*^	- ^a^	-
Non effusive	2	9.6 ± 0.05^b^	-
	3	10.73 ± 0.06^cdefg^	-
	6	10.41 ± 0.82^cde^	-
	14	10.25 ± 0.17^c^	-
	15	10.53 ± 0.01^cde^	-
	16	10.92 ± 0.34^cdefgh^	-
Effusive	4	11.06 ± 0.28^efghi^	N/A
	7	11.31 ± 0.33^ghi^	14.16 ± 0.05^l^
	8	10.74 ± 0.09^cdefg^	13.01 ± 0.04^k^
	9	11.72 ± 0.06^ij^	12.13 ± 0.18^j^
	5	11.28 ± 0.31^fghi^	13.21 ± 0.05^k^
	10	12.13 ± 0.35^j^	10.6 ± 0.65^cdef^
	11	10.99 ± 0.07^defgh^	10.31 ± 0.09^cd^
	12	11.57 ± 0.28^hij^	11.69 ± 0.19^ij^
	13	10.78 ± 0.74^cdefg^	12.02 ± 0.05^j^

*N/A* not available, *PES* supernatant of peritoneal effusion

^*^Cat 1 represents three healthy cats as negative controls

Note: Data are presented as means ± SEM of three replicates. Different alphabets denote significant difference (*p* < 0.05) following Duncan post hoc analysis

#### Expression profiles of immune-related genes in PBMC

The expression profiles of five immune-related genes, which were analyzed following in vitro infection of CRFK cells, were also analyzed in the PBMC and PE cells isolated from the FCoV-positive cats. In addition to normalization to GAPDH, the relative expression of the immune-related genes were normalized to the negative controls. As shown in Table 7, expression of all the genes except for CCL17 were detected in the PBMC of the sampled cats. However, gene expression levels varied among the cats. Most of the cats did not express or expressed very low levels of CCL8 and CXCL10 compared to healthy cats, except for cats 2, 3 and 5.

**Table 7:** Relative expression profiles of immune-related genes in PBMC of FCoV-positive cats

Cat status	Cat ID	CCL8 (MCP2)	CXCL10 (IP10)	MX1	Viperin (RSAD2)
Negative	1^*^	ND	ND	ND	1 ± 0^bc^
Non-effusive	2	ND	18.67 ± 0.33^b^	6.79 ± 1.8^e^	−1.88 ± 0.27^bc^
	3	16.32 ± 5.48^c^	ND	0.91 ± 0.1^cd^	ND
	6	ND	ND	0.03 ± 0^a^	−86.21 ± 0.01^a^
	14	0.42 ± 0.02^a^	ND	1.45 ± 0.36^d^	−28.01 ± 0.02^b^
	15	ND	ND	0.58 ± 0.04^c^	−94.34 ± 0.01^a^
	16	ND	ND	0.32 ± 0.07^b^	−39.84 ± 0.02^b^
Effusive	4	ND	ND	0.05 ± 0^a^	ND
	7	0.1 ± 0.05^a^	0.23 ± 0.22^a^	13.62 ± 5.07^fg^	4.87 ± 1.39^cd^
	8	ND	ND	3773.07 ± 67.71^j^	437.28 ± 31.23^f^
	9	ND	ND	7309.7 ± 52.55^k^	50.48 ± 3.44^e^
	5	1.08 ± 0.38^b^	19.17 ± 0^c^	19.14 ± 0.02^gh^	4.95 ± 0.31^cd^
	10	ND	ND	56.48 ± 13.47^i^	7.91 ± 5.25^de^
	11	ND	ND	6.79 ± 0.74^ef^	−3.35 ± 0.17^c^
	12	ND	ND	16.37 ± 5.13^g^	1.83 ± 0.45^bcd^
	13	ND	ND	26.9 ± 1.61^h^	10.16 ± 1.08^de^

*ND* Not detected

^*^Average expression of three healthy cats as negative controls. Relative expression (ΔΔCq) was calculated by normalizing to the reference gene (GAPDH) and negative controls

Note: Data are presented as means ± SEM of three replicates. Different alphabets indicate significant difference following Duncan post hoc comparison of each column (*p* < 0.05)

The expression of MX1 was detected in all FCoV-seropositive cats but not in healthy cats, and higher expression levels were detected in FCoV-positive cats with effusions (Table 7). Although viperin functions as an IFN-induced antiviral protein, similar to MX1, different patterns of viperin expression was observed. In addition, five out of six cats without signs of effusion showed downregulation of viperin compared to control cats (Table 7). Nevertheless, in cats with effusions, expression of viperin showed a trend similar that of MX1. In addition, a majority of the FCoV-positive cats with effusions showed markedly elevated expression levels of MX1 and viperin. As expected, the FCoV-negative cats did not express any of the analyzed immune-related genes, except for viperin.

#### Expression profiles of immune-related genes in peritoneal effusion cells

No distinct expression pattern was observed in the cellular component of PE collected from FCoV-positive cats (Table 8). However, high expression of CCL17 was detected in PE samples from three out of eight FCoV-positive cats with effusions. Meanwhile, cat 8, which showed the highest expression of CCL17, also exhibited the highest expression of MX1 and viperin as well. In addition, most of the cats that expressed MX1 also expressed viperin and CXCL10, suggesting the involvement of interferon-induced antiviral proteins; however, their expression levels varied significantly among different cats.

**Table 8:** Relative expression profiles of immune-related genes in cells from peritoneal effusion

Cat ID	CCL8 (MCP2)	CXCL10 (IP10)	CCL17	MX1	Viperin (RSAD2)
7	1.77 ± 0.29^d^	2.14 ± 0^e^	0.37 ± 0.19^bc^	1.65 ± 0.09^c^	2.14 ± 0^e^
8	0.29 ± 0.06^b^	0.57 ± 0.2^bc^	18.95 ± 16.6^cd^	11.24 ± 0.82^e^	13.31 ± 1.98^f^
9	0.46 ± 0.04^bc^	0.36 ± 0.1^b^	12.69 ± 6.01^d^	0.58 ± 0.02^b^	0.03 ± 0^a^
5	0.12 ± 0.02^a^	0.06 ± 0.01^a^	0.37 ± 0.18^cd^	0.61 ± 0.03^b^	0.34 ± 0.02^b^
10	1.43 ± 0.11^d^	0.64 ± 0.1^bcd^	0 ± 0^a^	1.36 ± 0.1^c^	0.79 ± 0.12^c^
11	0.1 ± 0.04^a^	0.85 ± 0.07^cd^	9.18 ± 3.87^d^	0.21 ± 0.03^a^	1.15 ± 0.13^d^
12	4.62 ± 0.08^e^	2.21 ± 0.38^e^	0.03 ± 0.03^ab^	4.7 ± 0^d^	1.97 ± 0.34^e^
13	0.79 ± 0.1^c^	1.04 ± 0.14^d^	1.83 ± 1.17^cd^	0.51 ± 0.05^b^	0.97 ± 0.07^cd^

Note: Data are presented as means ± SEM of three replicates. Different alphabets indicate significantly different groupings following Duncan post hoc comparison of each column (*p* < 0.05). Relative expression (ΔΔCq) was calculated by normalizing to the reference gene (GAPDH) and negative controls

#### Expression profiles of immune-related proteins in serum and peritoneal effusion supernatant

MILLIPLEX analysis of the serum and PES from the FCoV-positive cats revealed that all 19 immune-related proteins were detectable (Tables 9 and 10). However, no clear pattern was observed between the different levels of cytokines in cats with or without the presence of peritoneal effusions. Nevertheless, the expression of the immune-related proteins was higher in PES than in serum.

**Table 9:** Concentrations (pg/ml) of immune-related proteins in the serum of FCoV-positive cats

FIP Form	ID	Fas	Flt-3lL	GM-CSF	IFNγ	IL1β	IL2	PDGF-BB	IL12/p40	IL13	IL4	IL6	IL8	KC	SDF1	RANTES	SCF	MCP1	TNFα	IL18
NC	1^a^	<8.3 ± 0	45.3 ± 1.4	8.3 ± 0.1	12.3 ± 0	<17.3 ± 0	19.1 ± 0.2	300.1 ± 4.0	194.3 ± 5.3	5.6 ± 0	106.5 ± 1.7	<36.3 ± 0	13.0 ± 0.2	3.7 ± 0.2	92 ± 1.4	8.4 ± 0.2	118.3 ± 2.5	845.5 ± 0	115 ± 1.7	<71.2 ± 0
Dry	2	<8.3 ± 0	13.5 ± 0.2	7.6 ± 0.3	16.4 ± 0.2	<17.3 ± 0	17.1 ± 0.5	297.5 ± 4.5	1269 ± 11.6	5.9 ± 0.2	124.7 ± 1.4	<36.3 ± 0	32.6 ± 0.2	25.6 ± 0.9	64.8 ± 1	19.8 ± 0.1	49.0 ± 0.3	958 ± 12.5	65.0 ± 1.3	<71.2 ± 0
	3	9.8 ± 0.13	54.7 ± 1.7	15.0 ± 0.2	47.5 ± 0.4	<17.3 ± 0	17.8 ± 0.9	333.2 ± 0	268.5 ± 6.5	8.5 ± 0.1	287.4 ± 2.8	<36.3 ± 0	22.7 ± 0.5	25.7 ± 0.5	97.9 ± 0	19.3 ± 0.6	65.7 ± 2	1890 ± 0	152.3 ± 7.1	151.4 ± 9.8
	6	10.7 ± 0.1	24.9 ± 0.7	24.3 ± 0.1	39.2 ± 0.7	19 ± 0.94	24.8 ± 0	363.5 ± 3.3	147.6 ± 1.8	13.3 ± 0.4	323.2 ± 12.4	<36.3 ± 0	28.0 ± 0.6	22.8 ± 1.0	117.7 ± 0	8.6 ± 0.3	73.2 ± 2.3	1738 ± 41	104.28 ± 2	477.9 ± 5.7
Wet	7	9.1 ± 0.3	27.3 ± 2.4	15.2 ± 0.5	43.2 ± 2.5	<17.3 ± 0	17.8 ± 0.9	319.3 ± 8.1	543.9 ± 40.5	8.2 ± 0.3	117.3 ± 2.9	<36.3 ± 0	17.8 ± 1.6	3.3 ± 0.5	96.4 ± 2.6	16.8 ± 1.7	39.2 ± 3.7	914 ± 12.9	59.3 ± 2	<71.2 ± 0
	8	8.9 ± 0.1	14.1 ± 0.1	27.0 ± 0.3	14.2 ± 0.2	<17.3 ± 0	17.9 ± 0	339.6 ± 3.7	237.5 ± 0.5	6.2 ± 0	107.4 ± 2.9	<36.3 ± 0	19 ± 0.2	27.6 ± 0.7	85.9 ± 1.8	9.0 ± 0.2	<32.5 ± 0	868.6 ± 13.4	55 ± 0.5	<71.2 ± 0
	9	9.2 ± 0	39.5 ± 1.1	20.2 ± 0.1	61.9 ± 1.4	<17.3 ± 0	20.8 ± 0	332.8 ± 7.6	958.2 ± 36.8	7.8 ± 0	166.5 ± 0	<36.3 ± 0	16.0 ± 0.3	45.3 ± 1.9	84.3 ± 0.9	11.7 ± 0.3	39.5 ± 0.9	914.1 ± 12.9	66.3 ± 0	<71.2 ± 0
	5	11.5 ± 0.1	56.4 ± 0.1	16.2 ± 0.3	21.9 ± 0	<17.3 ± 0	22.9 ± 0.4	333.2 ± 0	2923.5 ± 70.7	7.1 ± 0.1	139.5 ± 1.4	<36.3 ± 0	28.9 ± 0.5	4.1 ± 0.1	90.5 ± 0.9	14.3 ± 0.1	46.6 ± 0.2	979.6 ± 0	88.8 ± 0	<71.2 ± 0
	10	13.5 ± 0.3	44.2 ± 0.8	13.8 ± 0.2	85.6 ± 2.5	32.6 ± 0.3	22.2 ± 0	305.3 ± 0	564.8 ± 5.3	10.4 ± 0.2	420.4 ± 10.9	116.7 ± 7.1	29.3 ± 0.2	3.3 ± 0.2	90.4 ± 4.4	22.8 ± 0.3	76.5 ± 1.5	2379 ± 13.3	158.2 ± 6.1	<71.2 ± 0
	11	12.6 ± 0	29.7 ± 0.9	19.1 ± 0.1	28.3 ± 1.2	<17.3 ± 0	26.0 ± 0	385.2 ± 3.0	1131 ± 17.3	11.2 ± 0.2	187.2 ± 6.3	<36.3 ± 0	37.6 ± 0.2	184.2 ± 7.0	189.3 ± 2.6	35.4 ± 0.6	69.2 ± 1.9	1082 ± 11.6	100 ± 2.5	96 ± 3.9
	12	12.2 ± 0	49.8 ± 1.7	11.1 ± 0.6	36.2 2.5	51.5 ± 6.9	95.3 ± 33.3	418.6 ± 5.2	655.1 ± 8.6	9.1 ± 0.3	294.4 ± 31.8	328.1 ± 56.2	28.9 ± 0.4	39.7 ± 34.8	303.9 ± 25.0	37.7 ± 0.4	48.9 ± 1.9	1370 ± 48.5	403.5 ± 40.6	522 ± 160.3

Note: Each sample was analyzed in three replicates and the data are expressed as means ± SEM

^a^Averaged concentrations from three healthy cats as negative controls (NC)

**Table 10:** Concentrations (pg/ml) of immune-related proteins in the PES of FCoV-positive cats

Cat ID	Fas	Flt-3lL	GM-CSF	IFNγ	IL1β	IL2	PDGF-BB	IL12/p40	IL13	IL4	IL6	IL8	KC	SDF1	RANTES	SCF	MCP1	TNFα	IL18
7	12.8 ± 0.4	226.1 ± 15.6	18.1 ± 0.4	5157 ± 503.5	54.2 ± 0.7	22.9 ± 0.4	357.5 ± 6.7	1033.6 ± 89.7	7.0 ± 0.2	125.9 ± 6.4	1123 ± 67.6	60.9 ± 5.5	4.9 ± 0.4	225.5 ± 3.1	650.5 ± 55.9	90.5 ± 3.1	968.7 ± 18.7	181.4 ± 2.4	<71.2 ± 0
8	9.6 ± 0	15.5 ± 0.5	23.8 ± 0.7	60.9 ± 1.2	82.7 ± 2.3	19.4 ± 0	319.7 ± 0	1040.9 ± 56.1	6.0 ± 0.1	109.9 ± 1.4	424.0 ± 11.4	308.3 ± 10.8	7.6 ± 0.6	220.7 ± 4	34.1 ± 1.5	44.0 ± 0.6	868.6 ± 13.4	56.7 ± 0.5	<71.2 ± 0
9	11.5 ± 0.4	218.3 ± 7.4	20.6 ± 0.3	5595 ± 250.6	54.8± 2.2	20.1± 0.4	345.5 ± 7.1	1598.5 ± 66.7	7.5 ± 0.2	149.3 ± 4.3	2998.5 ± 90.9	96.8 ± 3.3	12.1 ± 0.6	368.2 ± 14.3	180.1± 8.4	92.1± 2.9	968.7 ± 18.7	234.9 ± 9.1	<71.2 ± 0
5	10.0 ± 0.3	159.1 ± 6.2	17.4 ± 0.2	21.5 ± 1.1	21.3 ± 1	21.5 ± 0.4	319.7 ± 0	690.7 ± 29.7	7.0 ± 0.2	112.3 ± 2.9	96.2 ± 14.9	76.2 ± 3.3	4.1 ± 0.4	314.4 ± 4.6	27.2± 1.1	36.9 ± 1.7	868.6 ± 13.4	67.2 ± 1.5	<71.2 ± 0
10	11.1 ± 0.1	113.5 ± 1	15.9 ± 0.1	1072.5 ± 19.9	43.6 ± 0.5	20.1± 0.4	333.2 ± 0	908.8 ± 6.1	8.9 ± 0.1	382.6 ± 2.7	714.5 ± 1.4	86.8 ± 0.6	1.6 ± 0.1	372.4 ± 7.9	83.3 ± 0.7	82.8± 1	2228.5 ± 6.6	183.1 ± 0.5	<71.2 ± 0
12	13.7 ± 0.1	121.7 ± 4.4	10.3 ± 0.1	170.0 ± 3.1	128.8 ± 5.7	201.2 ± 8.2	380 ± 0	582.9 ± 8.6	10.8 ± 0	523.8 ± 8.1	1302 ± 61.2	46.7 ± 1.5	22.0 ± 0.8	1679± 66.4	64.6 ± 2.3	52.4 ± 0.6	1371.5 ± 9.5	988.1 ± 41.5	1412.5 ± 58.6
13	25.0 ± 0.1	133.6 ± 0.6	11.7 ± 0	1888.5 ± 22.8	242.3 ± 1.8	45.2 ± 0.2	521.1 ± 3.5	3708.5 ± 11.3	13.4 ± 0.2	2000.5 ± 2.6	6736.5 ± 13	303.6 ± 3.6	3.4± 0.1	206.1 ± 0.6	411.6 ± 5.3	36.6± 0.4	1268.5 ± 10.1	942.7 ± 10.9	194.0 ± 1.9

Note: Each sample was analyzed in three replicates and the data are expressed as means ± SEM

Although no common pattern of expression was seen among the FCoV-positive cats, detected levels of the different immune-related proteins in serum were higher in cats with peritoneal effusions compared to non-effusive FCoV-positive cats. The expression of pro-inflammatory cytokines and chemokines, such as GM-CSF, IFNγ, IL8, KC, RANTES, and MCP1, was readily detected in the serum of FCoV-positive cats (Table 9). The expression of IL1β and IL6 was not detected in the serum of the majority of the cats; however, these cytokines were detected in the PES of cats with peritoneal effusions (Table 10). TNFα production was detected in both FCoV-positive and negative cohorts. The production of this cytokine in the control cats could be due to an inflammatory process unrelated to FCoV infection, such as physiological stress [28]. In addition, the pro-inflammatory cytokine IL-18 was not consistently detected in both serum and PES. Unlike other immune-related proteins, serum levels of stem cell factor (SCF) were lower in the FCoV-positive cats compared to the control cats (Table 9).

## Discussion

Feline infectious peritonitis (FIP) is one of the leading causes of death among young cats [1]. Since FIP is an immune-mediated viral disease, studies using immunological approaches are crucial for a better understanding of the illness, particularly by using clinical samples of FIP cases before further studies utilizing experimental infection in cats could be justified. Detection of FCoV antigen in affected tissues by immunohistochemistry remains the gold standard in the confirmation of FIP [2].

One of the limitations of this study is that definitive confirmation of FIP was not made due to the unavailability of post-mortem samples. Therefore, the cats were selected based on their FCoV antibody and antigen status. In addition, the selected FCoV-positive cats were grouped according to the presence of peritoneal effusions at the time of clinical evaluation. Although we could not confirm the status of FIP in these cats, this study provides a preliminary examination on the array of immune mediators that may be involved in the development of FIP. In this study, more than 20 immune mediators were characterized following FIPV 79–1146 infection of CRFK cells and in FCoV-positive cats. Different expression profiles of immune mediators were detected in FIPV 79–1146-infected CRFK cells and those from FCoV-positive cats. Furthermore, the CRFK cells were used to optimize the real-time PCR detection of the different immune-related genes and to detect interferon-related genes during viral infection.

Based on an NGS transcriptomic study, we showed that pro-inflammatory and interferon-related genes, namely CCL8 (MCP2), CXCL10 (IP10), CCL17, MX1 and viperin (RSAD2), were upregulated in FIPV 79–1146-infected CRFK cells [14]. In this study, we confirmed the upregulation of these genes using Taqman real-time PCR (Table 2); however, detected levels of expression varied, which could be due to the differences in the sensitivity of these different platforms. One of the genes of interest that was highly upregulated and associated with an increase in viral load is MX1, an interferon-induced GTP-binding protein. Previous studies have shown that MX1 is an interferon-inducible protein found in humans and various animals that mediates resistance against RNA viruses [29]. In this study, we showed that mRNA expression of MX1 was significantly upregulated at 48 and 72 hpi and found to be correlated with the viral load at 48 hpi (Tables 2 and 3). Previous studies have also shown that the antiviral role of this gene is related to IFNα and β (IFN type 1) induction and GTPase pathways [19]. Similar to MX1, RSAD2, which is also known as viperin, is a gene that encodes for an IFN-induced antiviral protein [30]. However, unlike MX1, which is activated by type I IFN, viperin is induced by different types of IFN [31, 32]. In fact, the expression of viperin can be induced by double-stranded RNA analogs such as poly I:C, lipopolysaccharides and by infection with a broad range of both RNA and DNA viruses, indicating the diverse role of viperin during infection [31, 32]. The importance of this finding is not clear; nevertheless, studies have shown that viruses such as Japanese Encephalitis Virus (JEV) [33] and Dengue Virus type 2 (DENV-2) [34] can downregulate antiviral innate immune responses such as viperin and other IFN-inducible protein expression [31, 32]. Further studies are required to measure type I IFN levels in cats with FIP.

The clinical relevance of the observed variations in MX1 and viperin expression to the development of FIP is unknown. However, a study has shown that expression of viperin is crucial for optimal Th2 cell response in mice [35]. Hence, it is essential to further evaluate the importance of this finding in FIP cats. Earlier studies have proposed that the fundamental difference in the immune profiles of dry and wet forms of FIP is based on the predominant T cell responses. Cats with the dry form of FIP have a higher number of Th1 cells for the induction of CMI response, while cats with the wet form of FIP generally showed Th2-type response that leads to humoral immune response [13, 19].

The majority of FIP cases involve the presence of abdominal effusion, which was observed in eight out of 15 FCoV-positive cats sampled in this study [2]. Based on the pathogenesis of FIP, the accumulation of fluids in the peritoneal cavity of these cats is most probably due to the accumulation of the infected macrophages in the inter-venular space and venule walls [1, 10]. Activated and FCoV-infected monocytes can induce phlebitis through the paracrine and autocrine action of CD18, IL-1β and TNFα [11]. In addition, higher secretion of vascular endothelial growth factor (VEGF) has been associated with an increased production of effusion in cats with FIP [36]. Also, studies have shown that PE of cats diagnosed with FIP consisted primarily of macrophages and neutrophils with a low number of lymphocytes [37]. This study is in agreement with another study that reported a higher viral load in the supernatants of PE compared to those derived from the blood component of affected cats (Table 6) [15]. In addition, as expected, this study detected high expression of pro-inflammatory cytokines and chemokines, namely GM-CSF, IFNγ, IL8, KC, RANTES and MCP1, which are secreted mainly by monocytes/macrophages. The detection of CCL17 in the cell component of PE but not in PBMC indicated the inflammatory nature of the activated cells such as macrophages and dendritic cells (DC) present in the PE which may play an important role in the activation of Th2 cells [38, 39]. Furthermore, the lack of CCL17 expression, a chemokine that is primarily expressed in Th2 cells of cats with allergic inflammation [40], in the PBMC of the cats sampled in this study suggests a local rather than systemic response to the virus as also observed in other studies [17, 41, 42]. Further studies are warranted to confirm the expression of CCL17 by the activated cells from the PE of FIP cats. In addition, the downregulation of SCF (Table 9) is probably associated with the reduction of DC in cats with FIP, since SCF and Flt-3 L are important cytokines for the ex vivo propagation of human and mice DC [43, 44]. It was known that DC could be infected by FCoV; however, the role of SCF and Flt-3 L in FIPV infection warrants further examination [45].

In this study, the immune mediator protein levels vary between individual cats within the different cohorts (Tables 9 and 10). These findings were expected as biological individual variation could occur and has been observed in several other natural and experimental FIPV infections [12, 19]. Nevertheless, the expression of pro-inflammatory cytokines, namely IL1β and IL6, was more readily detected in the PES rather than the serum of the cats diagnosed with FIP. This finding is in agreement with previous studies that showed IL1 and IL6 can be detected in the serum and PE of FIP cases but not in those of healthy cats [18]. Besides, previous studies have shown that IL1β is related to CNS involvement and can only be detected in the inflammatory cells in the brain [19]. In this study, we found that IL-18, a pro-inflammatory cytokine in the IL-1 family that plays a major role in the activation of NK and T cells [46], was not readily detected in both serum and PES. The upregulation of TNFα protein in the serum and PES of some of the cats was in line with findings by previous studies that detected an increase in TNFα mRNA in abdominal effusions and PBMC of FIP-positive cats [14]. In addition, it has been suggested that this cytokine is responsible for T cell apoptosis [14, 21]. Hence, the role of these cytokines in FCoV-positive cats requires further evaluation. Interestingly, most of the FCoV-positive cats in this study have increased Fas serum levels, which may suggest a possible role of Fas in T cell apoptosis observed in FIP, as apoptosis can be induced by overexpression of Fas during viral infection [47].

## Conclusions

In conclusion, this study has established some insights on the different expression of immune mediators in FCoV-positive cats, where several immune mediators including pro-inflammatory cytokines, Th1-like cytokines, and IFN-related antiviral proteins were found to be highly expressed. In addition, no clear indication of Th1 and Th2 imbalance was detected in the various samples analyzed in this study. However, in general, MX1, viperin, CXCL10, CCL8, RANTES, KC, MCP1, IL8, GM-CSF and IFNγ were readily detected in FCoV-positive cats whereby MX1 and viperin expression was higher in FCoV-positive cats with peritoneal effusions. Future studies on FIP confirmed cases need to be carried out to further establish the importance of the different immune mediators in the development of FIP.

## Abbreviations

3’UTR: 3′ untranslated region
BLAST: Basic local alignment search tool
CMI: Cell-mediated immunity
CRFK: Crandell-Reese feline kidney
DC: Dendritic cell
FCoV: Feline coronavirus
FELV: Feline leukemia virus
FIP: Feline infectious peritonitis
FIPV: Feline infectious peritonitis virus
FIV: Feline immunodeficiency virus
hpi: Hours post inoculation
IACUC: Institutional animal care and use committee
NGS: Next-generation sequencing
PBMC: Peripheral blood mononuclear cell
PE: Peritoneal effusion
PES: Peritoneal effusion supernatant
qRT-PCR: Quantitative reverse transcriptase polymerase chain reaction
SARS: Severe acute respiratory syndrome
SPF: Specific-pathogen-free
SPSS: Statistical package for the social sciences
TCID_50_: Tissue culture infectious dose 50
UNG: Uracil-N-glycosylase
